# Acceptability of Cardiovascular Disease Point‐of‐Care Diagnostics in Primary Care Settings: A Scoping Review

**DOI:** 10.1002/hsr2.72032

**Published:** 2026-03-09

**Authors:** Nakedi Moswete, Siphesihle Robin Nxele, Penelope Modipane, Evans Duah, Bettina Chale‐Matsau, Gabrielle Thompson, Tivani Mashamba‐Thompson

**Affiliations:** ^1^ Department of Family Medicine, Faculty of Health Sciences University of Pretoria South Africa; ^2^ School of Health Systems and Public Health, Faculty of Health Sciences University of Pretoria South Africa; ^3^ Center for Development and Implementation of Point‐of‐care Diagnostics, Faculty of Health Sciences University of Pretoria South Africa; ^4^ School of Medicine, Faculty of Health Sciences University of Pretoria South Africa; ^5^ Department of Chemical Pathology, Faculty of Health Sciences University of Pretoria and National Health Laboratory Service South Africa; ^6^ Department of Geography and Sustainable Development, University of St Andrews University of St Andrews St Andrews UK

**Keywords:** acceptability, CVD, POCT, point‐of‐care, primary healthcare

## Abstract

**Background and Aims:**

Cardiovascular disease (CVD) remains the leading global cause of death, with 80% of deaths occurring in low‐ and middle‐income countries (LMICs). Improving access to screening and early diagnosis is essential. Point‐of‐care testing (POCT), which provides rapid results near the patient, is particularly valuable in resource‐limited settings. While POCT has been successfully implemented for infectious diseases like HIV and TB, investment in CVD‐focused POCT remains limited. This scoping review maps global evidence on the acceptability of CVD POCT, guided by the World Health Organization's REASSURED criteria, to inform future implementation strategies.

**Methodology:**

This review followed the Arksey and O'Malley framework. A comprehensive search was conducted across PubMed, Scopus, Science Direct, Google Scholar, Web of Science, and EBSCOhost databases. A preliminary search confirmed feasibility. Two reviewers independently screened studies at all stages, with agreement assessed statistically. The quality of included studies was appraised using the Mixed Methods Appraisal Tool (MMAT), version 2018.

**Results:**

Out of 738 articles identified, 13 primary studies conducted in primary care settings were included. Themes emerging from the review included POCT availability, influence on triage and clinical decision‐making, ease of use, sample volume, and feasibility. Only two studies were randomized controlled trials; the rest were observational, mostly comparing POCT accuracy to central laboratory testing. Inter‐reviewer agreement was high (Kappa = 0.92), and MMAT scores ranged from 71.4% to 85.7%.

**Conclusions:**

CVD POCTs are generally acceptable and demonstrate strong potential for clinical integration. However, a lack of robust evidence on patient outcomes, particularly from LMICs, limits the establishment of their effectiveness. More randomized controlled trials and economic evaluations in LMICs, where the burden of CVD is highest, are needed to support broader implementation and inform global strategies to reduce the impact of CVD.

## Summary

- What is known: CVD remains the leading cause of death globally, with limited use of POCT for its management in LMICs despite the success of POCT in infectious diseases.
- What is new: This scoping review identifies a significant gap in high‐quality evidence on the clinical utility and feasibility of CVD POCT, especially in LMIC primary care settings.
- What are the clinical implications: Expanding the use of CVD POCT in LMICs could improve early diagnosis and management, but implementation must be guided by robust, context‐specific evidence.

**Abbreviations**
ACS: acute coronary syndrome
ADHF: acute decompensated heart failure
cTn: cardiac troponin
CVD: cardiovascular disease
LMICs: low‐middle income countries
MMAT: mixed method appraisal tool
NT: proBNP: N‐terminal pro‐B‐type natriuretic peptide
PCC: population, concept, and context
POCT: point‐of‐care testing
PRISMA‐ScR: preferred reporting items for systematic reviews and meta‐analysis extension for scoping reviews
REASSURED: real‐time connectivity, easy to use, affordable, sensitive, specific, user friendly, rapid and robust, equipment‐free and deliverable to end‐users
SDG: Sustainable Development Goal
VTE: venous thromboembolism

## 1 Background

Cardiovascular disease (CVD) is one of the leading causes of death globally, with 80% of deaths occurring in low‐middle income countries (LMICs), with sub‐Saharan Africa as the most affected region [1]. The Global Burden of Disease Study 2019 (GBD 2019) reported that the global burden of CVD almost doubled between 1990 and 2019, with the number of people living with CVD rising from 271 to 523 million [2, 3]. Projections further indicate that between 2025 and 2050, global CVD prevalence will increase by 90.0%, resulting in an estimated 35.6 million CVD‐related deaths in 2050, up from 20.5 million in 2025 [4]. Improving access to CVD screening and monitoring can help improve health outcomes for the affected populations. Point‐of‐care testing (POCT) has been shown to be impactful in the prevention and monitoring of diseases in settings that have poor access to laboratory infrastructure. POCT is characterized by close proximity to the patient and relatively instant results without sample preparation, leading to fast clinical decisions [5]. In terms of noncommunicable diseases (NCDs), it has been shown to reduce the pressure on hospitals for monitoring CVD [6, 7].

Meanwhile, the United Nations’ Sustainable Development Goal 3 aims to ensure healthy lives and promote well‐being for all at all ages [7]. Accordingly, the World Health Organization (WHO) drafted a global action plan for the prevention and control of NCDs by advocating for primary healthcare access to essential diagnostics [7]. Notable accomplishments from the WHO's initiatives resulted in investments in decentralized POCT and treatment services in resource‐limited countries [7, 8, 9]. Thus, resulting in successful monitoring and management of communicable health conditions such as tuberculosis and human immunodeficiency virus (HIV) [7, 8, 9]. However, investment in decentralized POCT for monitoring NCDs, such as CVD, at the community‐level in resource‐limited settings is lacking [10]. Thus, testing gaps exist in resource‐limited settings, where populations carry the most burden of CVD [5, 10]. Resource‐limited communities depend heavily on public centralized healthcare for clinical diagnostics [7, 10, 11]. Consequently, a bottleneck is created in hospitals that potentially contributes to CVD mortality rates by delaying screening, diagnosis, and treatment for patients [6, 7, 10].

Clinical biochemistry POCT technology biomarkers of interest for CVD monitoring include cardiac troponins (cTn) [12], N‐terminal pro‐B‐type natriuretic peptide (NT‐proBNP) [13], and d‐dimers [14]. Studies were conducted in New Zealand [15] and Australian [16] rural areas where tertiary healthcare specialists remotely reviewed patients’ electrocardiograms and POCT cTn results to advise on management. These studies reported a decrease in missed acute coronary syndrome (ACS) diagnosis and improved rates of primary reperfusion therapy and 30‐day mortality, and also demonstrated safe and effective screening of ACS. A Netherlands study reported that about 30% decrease in NT‐proBNP levels during hospitalization predicted a favorable prognosis for patients with acute decompensated heart failure (ADHF) [17]. Furthermore, a meta‐analysis reported that d‐dimer POCT is significantly useful in guiding patient management and ruling out venous thromboembolism (VTE) in outpatients [18].

Evidence on acceptability of CVD POCT in primary care settings globally, particularly in LMICs is still unclear. This scoping review aims to systematically map evidence on the acceptability of CVD POCT in primary healthcare globally. In this study, acceptability was defined in accordance with the WHO's REASSURED criteria [19], which describe it as the extent to which a POCT is considered appropriate, user‐friendly, and satisfactory by both healthcare providers and patients. It includes factors such as ease of use, relevance in decision making, and the willingness of users and health systems to adopt and integrate into routine care. It is anticipated that the results of this study will identify gaps in the investment of resources in LMIC primary healthcare, inform future studies and interventions for implementation of POCT aimed at CVD in LMICs at the primary care level.

## 2 Methodology

### 2.1 Study Design

The protocol was developed following the Arksey and O'Malley scoping review methodological framework [20]. This framework involves a structured process that includes identifying the research question, searching for and selecting relevant studies, charting the extracted data, and finally collating, summarizing, and reporting the results. Furthermore, the quality of the included studies was appraised in accordance with the recommendations of Levac et al. [21]. This study followed reporting standards of the preferred reporting in systematic reviews and meta‐analysis (PRISMA) extension for scoping reviews (PRISMA‐ScR) [22]. Moreover, this scoping review protocol was registered on the Open Science Framework (OSF) (https://osf.io/493jx/).

### 2.2 Identifying the Research Question

The main research question guiding this scoping review was: What is the current state of evidence on the acceptability of CVD POCT technology in primary care settings globally? To determine the suitability of the question for the scoping review, the population, concept, and context (PCC) nomenclature (Table 1) recommended by the Joanna Briggs Institute was employed [23].

**Table 1:** PCC framework.

PCC element	Determinant
Population	Primary healthcare (PHC): PHC refers to an extensive range of health services provided by medical professionals in the community, for example, general practice (GP) surgeries, ambulances, community health centers, etc.
Concept	POCT assays used to detect and monitor patients for CVD: refers to CVD diagnostic testing near the patient using a platform, for example, cobas® h 232 (Roche), Atellica® VTLi (Siemens), PATHFAST® (LSI Medicine), TriageTrue® (Quide‐lOrtho), etc.
Context	Globally

### 2.3 Identifying Relevant Studies

A comprehensive literature search was conducted for relevant articles from PubMed, Scopus, Science Direct, Google Scholar, EBSCOhost (CINAHL, MEDLINE, Health Source/Nursing/Academic Edition, Academic Search Complete, and Open Dissertations), and Web of Science electronic databases. A limited range of the dates of publication between January 2000 and December 2024 was chosen. The search terms were in English. To find relevant studies, we searched for randomized controlled trials (RCTs), non‐RCTs, observational or evaluation studies, excluding review articles (systematic, scoping, narrative, meta‐analysis, and meta‐synthesis). In searching the databases, we used keywords such as “cardiovascular disease,” “point‐of‐care test,” “primary care,” “NT‐proBNP,” “d‐dimer,” “troponin,” “heart failure,” and “acute coronary syndrome,” combining terms with the Boolean operators AND and OR. We also examined the reference lists of included articles to identify additional relevant studies. An initial title screening was conducted, and all identified studies were imported into EndNote X9 (Bld 12062) for bibliographic management. Two reviewers independently screened the titles, abstracts, and full texts of all potentially relevant studies against the predefined eligibility criteria.

To determine the feasibility of this scoping review and identify relevant studies, a database search strategy was employed using different strings comprised of three or more search keywords. The results obtained from the pilot search of one of the selected databases are shown in the supplementary material (Suppinfo_S1). Strings including too few of the search keywords returned numerous results, but containing many that were irrelevant. Strings including more search keywords filtered and returned more specific results, although strings with too many of the search keywords returned no results. Hence, the search strings were adapted for each database to give a feasible number of results. The complete search output is presented in (Suppinfo_S2).

### 2.4 Study Selection

Eligibility criteria were developed to ensure that the selected studies contained relevant information to answer this scoping review's research question as follows:

### 2.5 Inclusion Criteria

- Articles which are primary studies
- Articles presenting evidence from PHC settings
- Articles that present evidence of CVD biomarker POCT usage
- Articles showing utilization of the following POCT platforms: cobas® h 232 (Roche), Atellica® VTLi (Siemens), PATHFAST® (LSI Medicine), and TriageTrue® (Quide‐lOrtho), and other relevant devices

### 2.6 Exclusion Criteria

- Articles without evidence of CVD POCT platform usage
- Articles with studies conducted before January 2000 and after December 2024
- Articles of POCT technology usage for health conditions other than CVD
- Articles which are reviews (systematic, meta‐analysis, scoping, etc.)
- Articles with studies conducted in secondary and tertiary healthcare settings.

### 2.7 Charting the Data

A standardized extraction sheet was developed and validated to extract data from included studies. The following information was extracted: author(s), year of publication, POCT platform, CVD marker(s), study setting, country, aim, study type, number of participants, and key findings.

### 2.8 Reviewer Agreement

Two reviewers independently screened all relevant study titles, abstracts, and full articles against the eligibility criteria. Both reviewers recorded their agreement (Yes/No) responses onto a word document (Suppinfo_S1), and it was used to calculate the Kappa statistic where values:

- ≤ 0 indicate no agreement
- 0.01–0.20 indicate none to slight agreement
- 0.21–0.40 indicate fair agreement
- 0.41–0.60 indicate moderate agreement
- 0.61–0.80 indicate substantial agreement
- 0.81–1.00 indicate almost perfect agreement

Thereafter, the statistical significance of the agreement between Reviewer 1 and Reviewer 2 was calculated using the McNemar significance value where a statistically significant result (*p* ≤ 0.05) indicates a significant difference in responses between the two reviewers, thus suggesting a lack of agreement (Suppinfo_S1). A nonsignificant result (*p* > 0.05) suggests an adequate level of agreement between the two reviewers, but it doesn't necessarily mean perfect agreement.

### 2.9 Quality Appraisal

An electronic version of the Mixed Method Appraisal Tool (MMAT) version 2018 was adapted and developed using Microsoft Excel to assess the quality of the included studies [24]. The MMAT allows for quality appraisal and description of methodological quality for three methodological category: mixed methods, qualitative, and quantitative (further subdivided into three sub‐domains: randomized controlled, non‐randomized, and descriptive). The quality of each of the selected studies was assessed according to the relevant methodological category in the tool (Suppinfo_S3). The MMAT was also used to examine the appropriateness of study aims, the context relevance, theoretical inferences to answer the research question, author discussions and conclusions. The overall quality for each of the included studies was calculated by following the MMAT guidelines (score = number of criteria met/total score in each category, expressed as a percentage). The results were presented using the following descriptors:

- Low quality (0%–25%), where minimal criteria are met
- Average (26%–50%)
- Above average (51%–75%)
- High quality (76%–100%), where all criteria are met

For mixed methods studies, the premise is that the overall quality of a combination cannot be more than the quality of its weakest component. Thus, the overall quality score would be the lowest score of the study components (qualitative or quantitative) [24].

### 2.10 Collating, Summarizing and Reporting the Results

The findings of this scoping review were summarized and reported using tables, graphs, and charts visualizing the key findings, and themes emerging from relevant and significant findings. Frequency distribution analysis was used to present variables of interest.

## 3 Results

### 3.1 Screening Results

**Figure 1:**
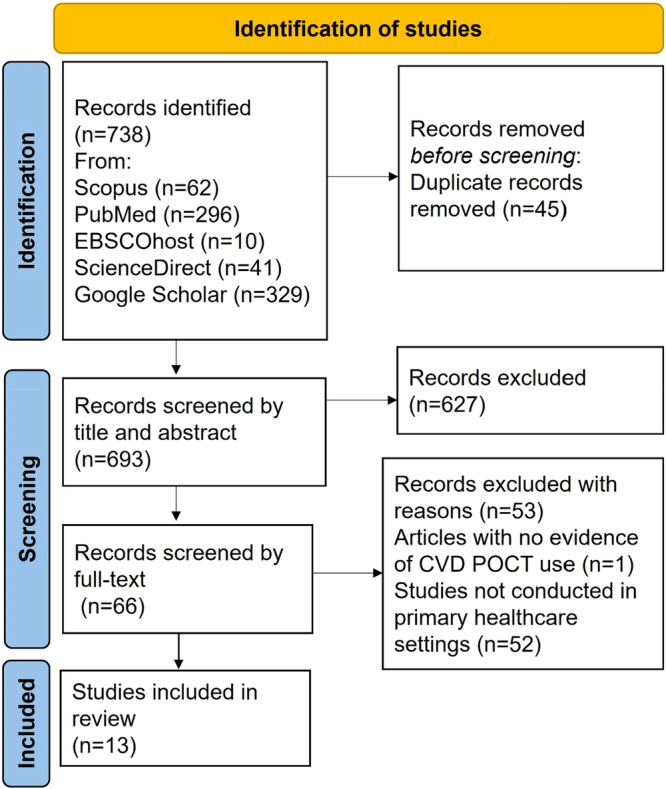
Preferred reporting items for systematic reviews and meta‐analysis (PRISMA‐ScR) flow chart.

The database search returned 738 articles. After the title and abstract screening by Reviewer 1 and Reviewer 2, 78 studies met the criteria to proceed to full‐text screening (Figure 1). After the full‐text screening, 13 articles met the inclusion criteria for this scoping review, and thus were proceeded to data extraction. A total of 627 articles were excluded at the abstract screening stage, and 53 articles were excluded in the full‐text screening stage with reasons based on the exclusion criteria. More specifically, 52 articles were excluded because they included studies which were not performed in primary care settings, and one article was excluded because it showed no evidence of CVD POCT usage (Figure 1). The agreement between the two reviewers after the full‐text article screening was assessed using the Kappa statistic and McNemar's statistic. The Kappa statistic (*K*) was equal to 0.91. Furthermore, the McNemar's statistic was 0.16 (*p* = 0.05).

### 3.2 Characteristics of the Included Studies

**Figure 2:**
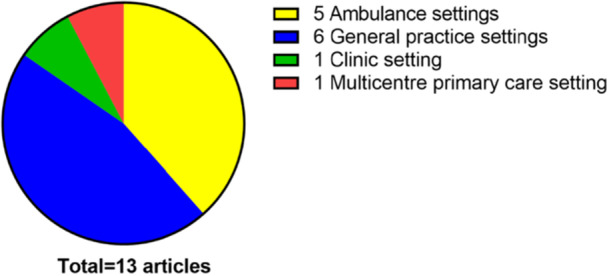
Distribution by primary care type for the selected articles.

**Figure 3:**
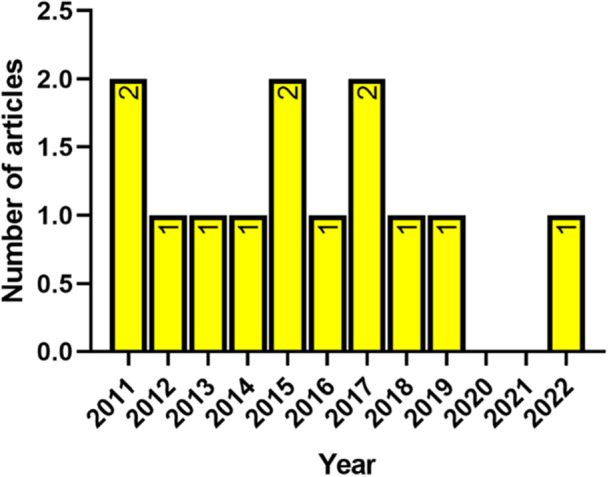
Distribution by year of publication.

**Figure 4:**
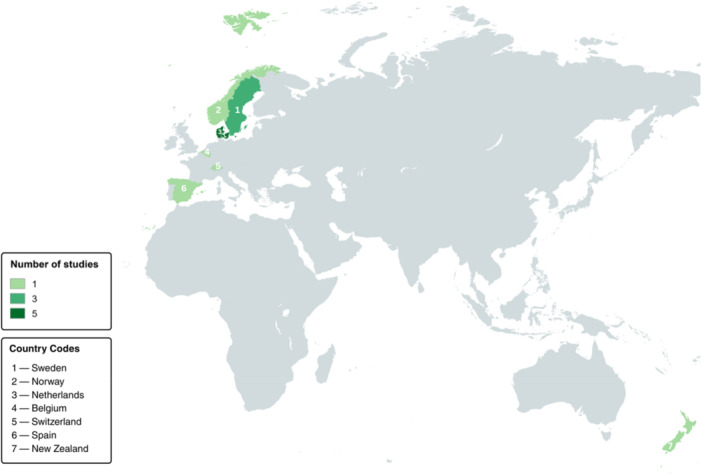
World map showing the distribution of the selected articles by country.

The characteristics of the selected articles are detailed in Table 2. All of the selected studies were conducted in primary care settings (Figure 2); with five of the studies conducted in an ambulance setting [25, 26, 27, 28, 29], six conducted in general practices [30, 31, 32, 33, 34, 35], and one study conducted in a clinic setting [36]. One of the studies was conducted in multiple primary care centers [37]. The selected studies were published between 2011 and 2022 (Figure 3). There were no selected articles which were published between 2000 and 2010. All of the selected articles were published in seven different countries (Figure 4). Five of the studies were conducted in Denmark [25, 26, 28, 29, 33], three of the studies were conducted in Sweden [31, 36, 37], and each of the rest were conducted in Spain [34], New Zealand [35], Belgium [30], Norway [27], and Switzerland [32].

**Table 2:** Characteristics and findings of the studies included in this scoping review.

Author(s)	Year of publication	Country	Study design	Study setting	No. of participant	Aim	POCT platform	CVD marker(s)	Key findings
Hex et al.[30]	2017	Belgium	Observational	General practice	94	Assess the accuracy of the Cobas h232 POC instrument in primary care versus hospital settings.	Cobas® h232 (Roche Diagnostics)	N‐terminal pro‐B‐type natriuretic peptide (NT‐proBNP)	The Cobas h232 NT‐proBNP test is accurate, easy to use, and beneficial in primary care.
Andersson et al. [31]	2015	Sweden	Observational	General practice	115	Evaluate high‐sensitivity troponin T in Swedish primary care and compare with POC troponin T results.	Cobas® h232 (Roche Diagnostics)	Troponin	High‐sensitivity troponin T POCT could be useful in primary care for those under 65, a higher threshold is needed for older patients.
Tomonaga et al. [32]	2011	Switzerland	Prospective observational	General practice	369	Analyse diagnostic accuracy of POCT in primary care.	Cardiac Reader® (Roche Diagnostics)	Troponin, NT‐proBNP and d‐dimer	POCT significantly improves correct diagnoses in primary care.
Khezri et al. [36]	2016	Sweden	Observational	Clinic	100	Evaluate Alere POC NT‐proBNP assay as a rapid alternative to lab testing in primary care.	i‐STAT Alinity (Abbott)	NT‐proBNP	The Alere NT‐proBNP test enables fast heart failure exclusion in primary care.
Gils et al. [33]	2015	Denmark	Observational	General practice	202	Compare Cobas h232 NT‐proBNP results in primary care with hospital lab results.	Cobas® h232 (Roche Diagnostics)	NT‐proBNP	The cobas h232 performs well in precision and ease of use
Rasmussen et al. [25]	2019	Denmark	Observational	Ambulance	16,449	Evaluate predictive value of routine prehospital POC troponin T for diagnosing suspected AMI.	Cobas® h232 (Roche Diagnostics)	Troponin	Elevated prehospital POC troponin T signals poor prognosis and aids early triage to advanced care.
Verdu et al. [34]	2012	Spain	Prospective observational	General practice	220	Determine optimal NT‐proBNP cut‐off in community primary care population.	Cobas® h232 (Roche Diagnostics)	NT‐proBNP	In primary care, NT‐proBNP cut‐off of 280 pg/mL effectively rules out heart failure.
Wells et al. [35]	2017	New Zealand	Randomized controlled observational	General practice	13,638	Assess the impact of POC testing for lipids and HbA1c on CVD risk assessments in general practice.	Cobas b 101 (Roche Diagnostics)	Lipids and HbA1c	Having a POC device within NZ general practices made no discernible difference to the completion of CVD risk assessments and was neither superior nor inferior to usual practice
Botker et al. [26]	2018	Denmark	Randomized controlled observational	Ambulance	747	Assess BNP testing by prehospital care teams for improved triage in severe dyspnea cases.	Cobas h232 (Roche Diagnostics)	NT‐proBNP	Adding NT‐proBNP to the physician diagnosis in prehospital dyspnea cases did not improve decision‐making or patient outcomes
Jacobssen et al. [27]	2022	Norway	Prospective observational	Ambulance	253	Assess feasibility and diagnostic accuracy of prehospital ECG, TnT, and TTE for early NSTEMI identification.	Cobas h 232 (Roche Diagnostics)	Troponin	Prehospital ECG, TnT, and TTE by paramedics can identify most NSTEMI cases early.
Nilsson et al. [37]	2014	Sweden	Observational	Primary care centers	196	Evaluate safety and cost‐effectiveness of POCT‐TnT for chest pain management in primary care.	N/A	Troponin	POCT‐TnT in primary care may reduce costs but with risk of missing some cases.
Sorensen et al. [28]	2011	Denmark	Observational	Ambulance	958	Assess prehospital TnT POCT feasibility and utility in improving chest pain diagnosis.	Cobas h 232 (Roche Diagnostics)	Troponin	Prehospital TnT testing is feasible and identifies AMI cases regardless of ECG.
Stengaard et al. [29]	2013	Denmark	Prospective observational	Ambulance	985	Study feasibility and utility of prehospital POCT for AMI and mortality prediction.	Cobas h 232 (Roche Diagnostics)	Troponin	Large‐scale prehospital cTnT POCT is feasible and predicts mortality.

**Figure 5:**
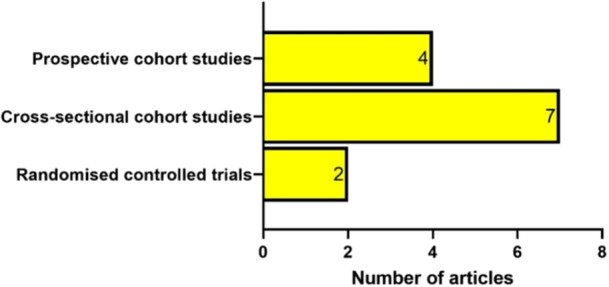
Distribution by study type for the selected articles.

All of the 13 selected articles were observational studies (Figure 5); two of them were RCTs [26, 35], seven of them were cross‐sectional cohort studies [25, 30, 31, 33, 36, 37], and four of the studies were prospective cohort studies [27, 28, 29, 32, 34].

**Figure 6:**
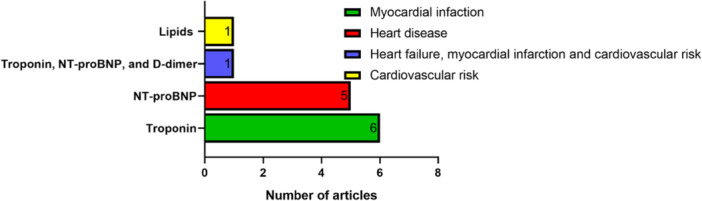
Distribution by CVD and corresponding biomarkers under investigation.

With regards to the type of CVDs under investigation in all of the 13 selected studies (Figure 6), six of them were aimed at troponin—a biomarker for myocardial infarction [25, 27, 28, 29, 31, 37], five of the studies were aimed at NT‐proBNP—a biomarker for heart failure [26, 30, 33, 34, 36], one of the studies was aimed at blood lipids—a biomarker for CVD risk [35], and one study concurrently measured d‐dimer, troponin, and NT‐proBNP; thus, adding d‐dimer—a biomarker for VTE to NT‐proBNP and troponin testing [32].

**Figure 7:**
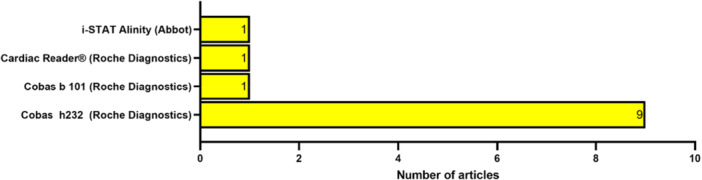
Distribution by CVD POCT platforms used in the selected articles.

The POCT platforms used in the selected studies are shown in Figure 7. In total, there were four different tests/devices utilized in all of the 13 selected studies, 9 of the studies utilized the cobas h232 device (Roche Diagnostics) [25, 26, 27, 28, 29, 30, 31, 33, 34], one of the studies utilized the cobas b101 instrument (Roche Diagnostics) [35], one of the studies utilized the cardiac reader device (Roche Diagnostics) [32], and one study utilized the i‐STAT Alinity (Abbot) [36]. One of the selected studies—a multicentre study, did not explicitly name the POCT platform which was utilized, although it heavily implied that at least one was indeed used to collect the data [37].

### 3.3 Quality of Included Studies

The quality appraisal scores of the 13 included articles averaged a value of 84.6% (standard deviation = 3.97). With a minimum score of 71.4%. [26] and a maximum score of 85.7% [25, 27, 28, 29, 30, 31, 32, 33, 34, 35, 36, 37].

### 3.4 Key Findings

The following themes regarding CVD POCT were extracted from the selected articles: availability in primary care centers, influence on triage and clinical decisions, ease of use, feasibility, and sample volume required. Some of the themes overlapped in one or more of the selected articles for this scoping review.

### 3.5 Availability in Primary Care Centers

All of the 13 selected articles reported evidence on the availability of CVD POCT diagnostics in primary care settings globally [25, 26, 27, 28, 29, 30, 31, 32, 33, 34, 35, 36, 37]. This scoping review reveals that clinical research on CVD POCT diagnostics has been disproportionately concentrated in secondary and tertiary care settings, with primary care largely underrepresented (Figure 1). Over the past 24 years, studies in higher‐level healthcare settings outnumbered those in primary care by approximately fourfold. Furthermore, although the review included studies from across the globe, all were conducted in high‐income countries, with none from LMICs, as classified by the World Bank (Figure 4) [38].

### 3.6 Influence on Triage and Clinical Decisions

Ten articles reported evidence on CVD POCT influence on triage and clinical decisions [26, 27, 28, 29, 30, 31, 32, 34, 35, 36]. Wells et al. [35] aimed to assess the impact of POCT for lipids and HbA1c on CVD risk assessments and reported that having a CVD POCT device made no significant difference to the completion of CVD risk assessments, and was no better or worse compared to standard clinical practice. Andersson et al. [31] aimed to evaluate high‐sensitivity troponin T and compared with POC troponin T results. They reported that a high‐sensitivity troponin T POCT could be useful in identifying cardiac events for those under 65, and a higher threshold is needed for older patients. Tomonaga et al. [32] aimed to analyse the diagnostic accuracy of CVD POCT and reported that CVD POCT significantly improves patients' diagnosis. Khezri et al. [36] aimed to evaluate CVD POCT as a rapid alternative to laboratory testing and reported that the CVD POCT enables fast heart failure exclusion. Verdú et al. [34] aimed to determine the optimal NT‐proBNP cut‐off using POCT and reported that CVD POCT effectively rules out heart failure. Hex et al. [30] aimed to assess the accuracy of the cobas h232 POC instrument and reported that CVD POCT is accurate and beneficial for heart failure diagnosis. Jacobsen et al. [27] aimed to evaluate the predictive value of routine POCT for diagnosing suspected myocardial infarction and reported that CVD POCT signals poor prognosis and aids early triage to advanced care. Bøtker et al. [26] aimed to assess CVD POCT for improved triage in severe dyspnea cases and reported no improvement in decision‐making or patient outcomes from adding POCT to physician‐based diagnosis for dyspnea. Stengaard et al. [29] aimed to study the diagnostic utility of prehospital POCT for myocardial infarction and mortality prediction and reported that large‐scale prehospital cTnT POCT data succesfully predict mortality. Sørensen et al. [28] aimed to assess prehospital POCT utility in improving chest pain diagnosis and reported that CVD POCT identifies myocardial infarction cases regardless of ECG. With regards to the limitations identified in the literature, only studies by Wells et al. [35] and Bøtker et al. [26] were RCTs. The rest of the eligible articles were non‐randomized observational studies. It goes without saying that RCTs are superior compared to other observational studies in that they minimize bias by ensuring that the observed differences in outcomes between the tested groups and non‐tested groups are most likely due to the intervention, rather than pre‐existing differences in the groups [39]. Hence, the results of this study highlight potential concerns in the literature with regards to the lack of empirical evidence in favor of CVD POCT over standard clinical practice in clinical situations.

### 3.7 Ease of Use

Two articles reported evidence on CVD POCT ease of use [30, 33]. Hex et al. [30] reported that the cobas h232 CVD POC instrument is easy to use. Gils et al. [33] reported that the cobas h232 CVD POCT device performs acceptably well and is easy to use. This scoping review identified a gap in the evidence for the ease of use of the CVD POCT presented in the literature. The results of this study show that although some of the selected articles reported ease of use, it should be noted that none of the studies directly assessed ease of use for the CVD POCT technology using a standardized methodology, such as the system usability scale. Limited available information on the ease of use of the CVD POCT devices may raise potential concerns for stakeholders who are interested in implementation. Therefore, this presents a gap in the literature which warrants further investigation in future research.

### 3.8 Feasibility

Three articles reported evidence on CVD POCT feasibility [28, 29, 37]. Nilsson et al. [37] aimed to evaluate the safety and cost‐effectiveness of POCT for patient chest pain management in primary care and reported that POCT in primary care may reduce costs but with the risk of missing some cases. Stengaard et al. [29] aimed to study the feasibility of POCT for myocardial infarction and mortality prediction and reported that large‐scale CVD POCT is feasible. Sørensen et al. [28] aimed to assess POCT feasibility in improving chest pain diagnosis and reported that CVD POCT is feasible. This scoping review identified a limited empirical evidence with regards to feasibility in the literature. Although positive feasibility is reported in some of the selected articles, it should be noted that none of the eligible articles in this scoping review provided in‐depth economic analysis, nor were they detailed feasibility studies, as it was not their intended purpose. This may raise concerns for stakeholders interested in implementation. Hence, this presents another gap in the literature which warrants thorough economic feasibility analysis in future research.

### 3.9 Sample Volume Required

A total of 12 selected articles reported evidence on the required sample volume for CVD POCT [25, 26, 27, 28, 29, 30, 31, 32, 33, 34, 35, 36, 37]. Nine articles which used the cobas h232 device required a maximum of 150 µL of sample [25, 26, 27, 28, 29, 30, 31, 33, 34]. One article, which used the i‐STAT Alinity device required a maximum of 100 µL [36]. One article which used the cobas b101 device required a maximum of 20 µL [35]. One article which used the cardiac reader device required a maximum of 150 µL of sample [32]. With regards to gaps identified in the literature, one article which was a multicentre primary care observational study implied CVD POCT usage but did not explicitly mention the POCT platforms that were used nor the amount of sample volumes used [37].

## 4 Discussion

This scoping review aimed to systematically map evidence on the use of CVD POCT in primary care settings globally. Herewith, the results highlighted the lack of research aimed at CVD POCT in LMICs at the primary healthcare level. This scoping review highlights the gap which exists in literature where research fails to address one of the core principles of the REASSURED criteria, that is deliverability to end‐users. LMICs have limited access to the potential benefits that come with POCT clinical research aimed at addressing CVD, a disease which disproportionately affects LMICs globally [1]. Furthermore, the notable lack of resources into LMIC clinical research is hindering the progress toward achieving Sustainable Development Goal (SDG) 3 which aims to promote good health and well‐being for all [40].

The lack of empirical evidence in favor of CVD POCT utility (e.g., predictive values, triage benefits, etc.) and feasibility (e.g., cost, user‐friendliness, etc.) in clinical situations over standard practice is hindering the large‐scale implementation at all levels of healthcare globally. Hence, this scoping review highlights a gap in the literature where research is failing to address the following core principles of the REASSURED criteria: affordability, sensitivity and specificity, user‐friendliness, and deliverability to end users. The literature aligned well with one of the core principles of the REASSURED criteria, ease of specimen collection, consistently demonstrating that very small sample volumes were required from patients.

Although the results of this scoping review show that the CVD POCT diagnostics market is well established and there is abundant research utilizing POCT platforms aimed at CVD. This scoping review identified a lack of research aimed CVD POCT diagnostics in LMICs. This is corroborated by a scoping review by Moore et al. [41] on POCT in emergency services globally, which reported that all of the articles included in their scoping review were conducted in high‐income countries as well, and none were conducted in LMICs. Furthermore, a scoping review by Moetlhoa et al. [42] reported that there was limited published research on POCT diagnostics in sub‐Saharan Africa, a region known to be comprised of LMICs [42]. Furthermore, this scoping review identified a lack of empirical evidence in the form of RCTs and feasibility studies or economic analysis, aimed at convincing clinicians that CVD POCT technology is beneficial to patients over standard clinical practice. This is corroborated by a 2025 scoping review by Moore et al. [41] which was not specific to CVD but broader implementation of POCT. The review reported lack of studies, including RCTs, that influenced or informed the usage of POCT over standard practice in clinical care. Furthermore, the authors reported lack of studies on economic analysis or the feasibility of POCT [41]. However, in alignment with our scoping review, Moore et al. [41] reported that majority of the articles in their scoping review consisted of observational studies which aimed to assess the accuracy of the POCT technology in comparison to standard central laboratory testing, that is, diagnostic accuracy studies.

Though diagnostic studies are important, they are not designed to answer the question whether CVD POCT is beneficial to patients over standard clinical care. This is further substantiated by a systematic review by Lingervelder et al. [43] on point of care testing in primary care, which reported that despite the growing market and development of new POCTs, observational studies evaluating the accuracy of the tests fail to report on aspects that clinicians also find important, such as whether POCT is beneficial to patients over standard primary care in clinical practice [43]. Furthermore, the authors suggested that future POCT evaluations should not only focus on the accuracy and comparability but also report on the aspects relating to the clinical utility and risks to patients, in order to ensure that a POCT is useful to primary care clinicians. This is in line with deliverability to end users, as clinical care providers would not be convinced to adopt POCT over standard practice if the utility of POCT to patient care is not proven beyond a reasonable doubt [43]. Hence, this further highlights the gap which needs to be filled by more randomized clinical trials, and ultimately an appropriate meta‐analysis.

## 5 Strengths and Limitations

As per standard practice, this scoping review utilized the Arksey and O'Malley scoping review methodological framework [20]. And the quality of the included studies was appraised as per Levac et al. recommendations [21]. Furthermore, this study followed reporting standards of the PRISMA extension for scoping reviews [22]. For article screening, the online tool Rayyan (https://www.rayyan.com/) was used to identify and remove duplicates and to facilitate screening at all stages. The screening in its entirety was blinded and included two reviewers, Reviewer 1 and Reviewer 2. The level of agreement between the two independent reviewers attained a Kappa statistic between 0.81 and 1.00, which indicates almost perfect agreement. Furthermore, the high level of agreement between the two reviewers was corroborated by the McNemar's *p*‐value, which was greater than the significance level (*p* < 0.05), meaning that there was no significant difference between the responses from the two reviewers. This indicates that this scoping review's methodology was rigorous and robust. Furthermore, 12 of the 13 selected articles in this scoping review attained a quality appraisal score of 85.7% which is deemed high quality, and only one article scored 71.4% which is deemed above average quality. Therefore, this scoping review on average used high‐quality sources. Despite attempts to be as comprehensive and thorough as possible with the literature search, there may have been other published articles and grey literature which may have been missed due to the search strings employed in each database and human error.

## 6 Recommendations for Practice and for Research

CVD POCT should be integrated into primary care in LMICs using tools that meet the REASSURED criteria to ensure suitability for resource‐limited settings. Implementation should be supported by provider training, supply chain readiness, and alignment with existing care pathways to enhance early detection and management of CVD. While observational studies assessing the diagnostic accuracy of CVD POCT relative to central laboratory methods are informative, they currently predominate the evidence base. However, this focus should be balanced with greater investment in RCTs, which offer more robust empirical evidence on clinical utility and patient outcomes. The generation of high‐quality RCT data is essential to support meta‐analysis and strengthen the evidence base for the effectiveness of CVD POCT in routine care. Furthermore, comprehensive economic evaluations and feasibility studies on CVD POCT in primary care are crucial to inform and engage policymakers in LMICs regarding potential implementation.

## 7 Conclusion

This scoping review highlights a significant imbalance in the research landscape for CVD POCT diagnostics, with limited progress in LMICs at the primary care level compared to high‐income countries. While global research on POCT has expanded, primarily focusing on diagnostic accuracy relative to central laboratory testing, a critical gap in assessing the clinical utility and associated risks of CVD POCT using RCTs remains. Additionally, feasibility studies on patient outcomes and influence on clinical decisions, and economic evaluations are lacking, yet essential to support implementation in resource‐limited settings. Although the REASSURED criteria provide a valuable framework for guiding innovation, efforts to synthesize evidence through meta‐analysis are constrained by the limited availability of high‐quality primary research and the frequent exclusion of populations most affected by CVD, particularly those in LMICs. Addressing these gaps is essential to promote health equity and support global efforts toward eliminating CVDs.

## Author Contributions

Tivani Mashamba‐Thompson and Nakedi Moswete conceptualized and designed the study. Nakedi Moswete prepared the initial draft of the manuscript. Siphesihle Robin Nxele, Evans Duah, and Penelope Modipane contributed to the screening of abstracts and full texts and performed the quality assessment. Gabrielle Thompson and Nakedi Moswete supported data presentation and visualization. Tivani Mashamba‐Thompson and Bettina Chale‐Matsau provided study supervision, validated the data extraction forms, and reviewed manuscript drafts. All authors have read and approved the final version of the manuscript. Evans Duah, the corresponding author, had full access to all of the data in this study and takes complete responsibility for the integrity of the data and the accuracy of the data analysis.

## Funding

The authors received no specific funding for this work.

## Ethics Statement

This study was conducted as a scoping review and did not involve human participants or the collection of primary data. All data were obtained from publicly available sources, and therefore, ethical approval was not required. The review was conducted in accordance with established methodological frameworks and reporting guidelines.

## Conflicts of Interest

The authors declare no conflict of interest.

## Transparency Statement

The lead author Evans Duah affirms that this manuscript is an honest, accurate, and transparent account of the study being reported; that no important aspects of the study have been omitted; and that any discrepancies from the study as planned (and, if relevant, registered) have been explained.

## Supporting information


**Suppinfo_1:** Pilot search and Inter‐rater agreement.

**Suppinfo_2:** Search strategy and output.

**Suppinfo_3:** Quality appraisal.

## Data Availability

The authors confirm that the data supporting the findings of this study are available within the article and its supplementary materials.
