# Supplementary material for: Acceptability of Cardiovascular Disease Point‐of‐Care Diagnostics in Primary Care Settings: A Scoping Review
Source: Health Sci Rep. 2026 Mar 9;9(3):e72032. doi: 10.1002/hsr2.72032 (PMC12971386; doi:10.1002/hsr2.72032)
Supplement: Supplementary file 1 — Suppinfo_1: Pilot search and Inter‐rater agreement. [file HSR2-9-e72032-s002.pdf]

Scoping review supplementary material

Part 1: Pilot database search results from PubMed search engine.

Keyword search	Date of search	No. of publications retrieved
("point of care systems"[MeSH Terms] OR ("point of care"[All Fields] AND "systems"[All Fields]) OR "point of care systems"[All Fields] OR ("point"[All Fields] AND "care"[All Fields]) OR "point of care"[All Fields]) AND ("research design"[MeSH Terms] OR ("research"[All Fields] AND "design"[All Fields]) OR "research design"[All Fields] OR "test"[All Fields] OR ("system"[All Fields] OR "system s"[All Fields] OR "systems"[All Fields])) AND ("heart diseases"[MeSH Terms] OR ("heart"[All Fields] AND "diseases"[All Fields]) OR "heart diseases"[All Fields] OR ("heart"[All Fields] AND "disease"[All Fields]) OR "heart disease"[All Fields]) AND ("pro brain natriuretic peptide 1 76"[Supplementary Concept] OR "pro brain natriuretic peptide 1 76"[All Fields] OR "nt probnp"[All Fields] OR ("troponin t"[MeSH Terms] OR "troponin t"[All Fields]) OR ("fibrin fragment d"[Supplementary Concept] OR "fibrin fragment d"[All Fields] OR "d dimer"[All Fields]))	12 February 2025	296

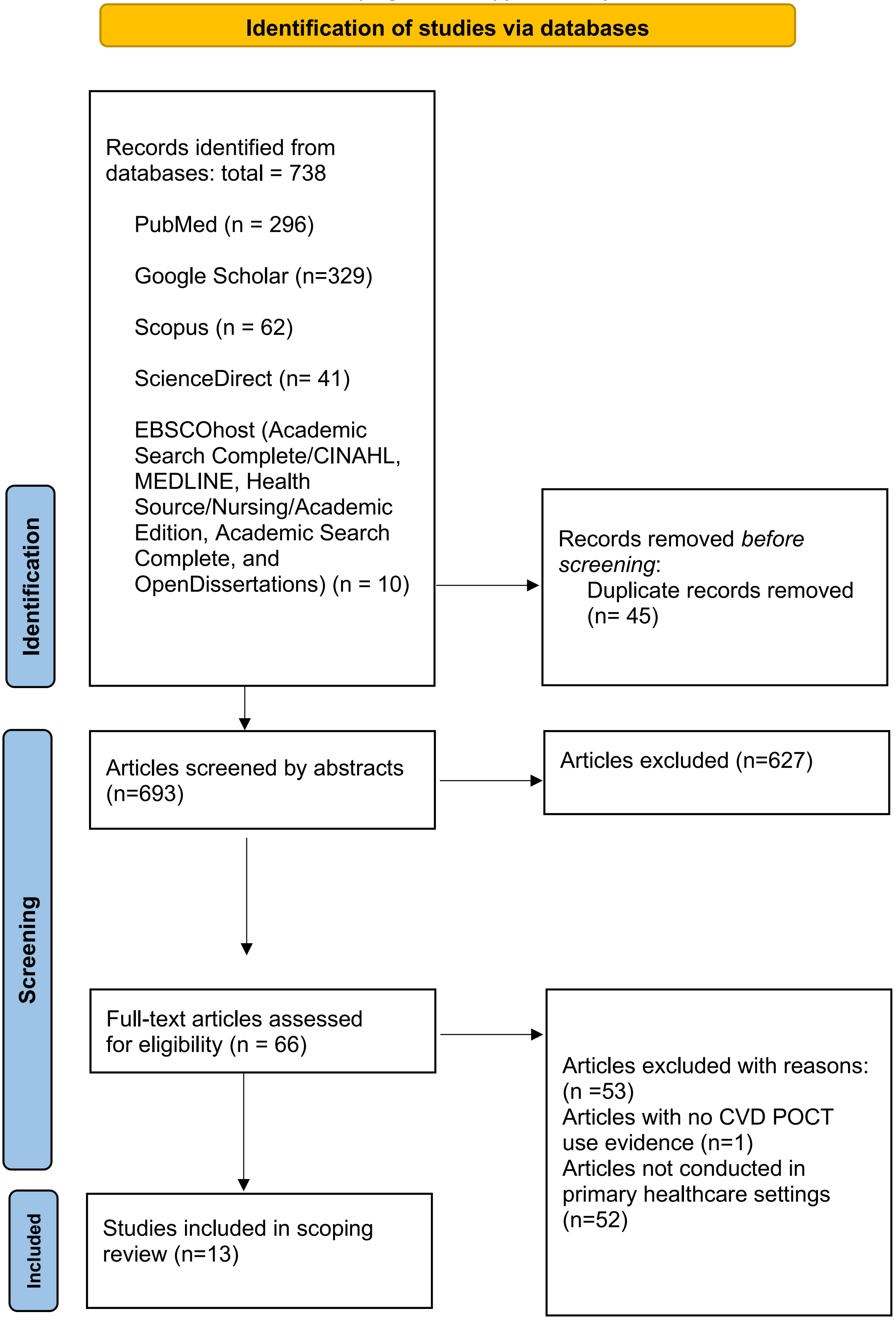

Part 2: PRISMA flow chart for the scoping review. Online software tool RAYYAN was used to identify and remove duplicates, as well as to screen the articles at all levels. The screening was blinded and included two reviewers: TM and ED.

Scoping review supplementary material

Part 3: Screening agreement results between the two independent reviewers.

Author and year	Reviewer 1 TM(Y/N)	Reviewer 2 ED(Y/N)
Mohammadzadeh et al. 2022	N	N
Toprak et al. 2024	N	N
Wells et al. 2017	Y	Y
Agewall et al. 2003	N	N
Alehagen et al. 2008	N	N
Alghamdi et al. 2020	N	N
Andersson et al. 2015	Y	Y
Asha et al. 2015	N	N
Bank et al. 2015	N	N
Bertsch et al. 2010	N	N
Birkhan et al. 2012	N	N
Botker et al. 2018	Y	Y
Calzavacca et al. 2012	N	N
Castro-Portillo et al. 2022	N	N
Ceriello et al. 2023	N	N
Chang et al. 2022	N	N
Collinson et al. 2013	N	N
Collinson et al. 2004	N	N
Cramer et al. 2007	N	N
Cruiset et al. 2008	N	N
Dupuy et al. 2015	N	N
Gils et al. 2015	Y	Y
Goldmann et al. 2004	N	N
Hallani et al. 2005	N	N
Hamilton et al. 2008	N	N
Hex et al. 2017	Y	Y
Higa et al. 2016	N	N
Iosifov et al. 2024	N	N
Iwasaki et al. 2017	N	N
Jacobssen et al. 2022	Y	Y
Jungbauer et al. 2012	N	N
Kellens et al. 2016	N	N
Khezri et al. 2016	Y	Y
Koechlin et al. 2024	N	N
Koechlin et al. 2024	N	N

Kratz et al. 2002	N	N
Lee-Lewandrowski et al. 2007	N	N
Lee-Lewandrowski et al. 2011	N	N
Leung et al. 2017	N	N
Li et al. 2010	N	N
MacDonald et al. 2008	N	N
Mad et al. 2007	N	N
Muller et al. 2022	N	N
Muller-Bardorff et al. 2000	N	N
Muresan et al. 2021	N	N
Nilsson et al. 2014	Y	Y
Ordonez-Llanos et al. 2006	N	N
Prosen et al. 2011	N	N
Rasmussen et al. 2019	Y	Y
Slagman et al. 2016	Y	N
Sorensen et al. 2011	Y	Y
Sorensen et al. 2019	N	N
Stengard et al. 2013	Y	Y
Suzuki et al. 2018	N	N
Tanaka et al. 2006	N	N
Avest et al. 2016	N	N
Tomonaga et al. 2011	Y	Y
Tsui et al. 2019	N	N
Vefaie et al. 2015	N	N
Van Dongen et al. 2018	N	N
Van Dongen 2020	N	N
Verdu et al. 2012	Y	Y
Wang et al. 2015	Y	N
Wilke et al. 2017	N	N
Willemsen et al. 2015	N	N
Zugck et al. 2006	Y	Y

Part 4: STATASE18 Kappa statistic output for reviewer agreement.

Agreement	Expected agreement	Kappa	Std. err.	Z	Prob>Z
96.97%	64.83%	0.9138	0.1226	7.45	0.0000

Part 5: STATASE18 McNemar's statistic output for reviewer agreement.

Cases	Controls Exposed	Controls Unexposed	Total
Exposed	14	2	16
Unexposed	0	50	50
Total	14	52	66

McNemar's chi2(1) = 2.00 Prob > chi2 = 0.1573
Exact McNemar significance probability = 0.5000

Proportion with factor

		[95% conf. interval]		
Cases	.2424242			
Controls	.2121212			
difference	.030303	-.0262044	.0868104	
ratio	1.142857	.9496443	1.375381	
rel. diff.	.0384615	-.0138073	.0907304	
odds ratio	.	.1878091	.	(exact)
